# Comparison of two laboratory-developed PCR methods for the diagnosis of Pulmonary Tuberculosis in Brazilian patients with and without HIV infection

**DOI:** 10.1186/1471-2466-11-15

**Published:** 2011-03-29

**Authors:** Luciene C Scherer, Rosa D Sperhacke, Carla Jarczewski, Patrícia I Cafrune, Candice T Michelon, Rubia Rupenthal, Marta Osorio Ribeiro, Antonio Ruffino Netto, Maria LR Rossetti, Afrânio L Kritski

**Affiliations:** 1 Post Graduation Program in Biological Science-Biochemistry Department, Federal University of Rio Grande do Sul-UFRGS, Porto Alegre/RS/Brazil; 2 Technological and Scientific Development Center-CDCT, State Foundation in Production and Health Research - FEPPS/RS) Porto Alegre/RS/Brazil; 3 Lutheran University of Brasil-ULBRA, Canoas/RS/Brazil; 4 Academic Tuberculosis Program, Medical School, Clementino Fraga Filho Hospital, ATP-MS/HUCFF, Federal University of Rio de Janeiro-UFRJ, Rio de Janeiro, Brazil; 5 Parthenon Hospital/Secretary of Health of Rio Grande do Sul/Porto Alegre/RS/Brazil; 6 Public Laboratory of the State of Rio Grande do Sul (LACEN/RS), State Foundation in Production and Health Research - FEPPS/RS) Porto Alegre/RS/Brazil

## Abstract

**Background:**

Direct smear examination with Ziehl-Neelsen (ZN) staining for the diagnosis of pulmonary tuberculosis (PTB) is cheap and easy to use, but its low sensitivity is a major drawback, particularly in HIV seropositive patients. As such, new tools for laboratory diagnosis are urgently needed to improve the case detection rate, especially in regions with a high prevalence of TB and HIV.

**Objective:**

To evaluate the performance of two *in house *PCR (Polymerase Chain Reaction): PCR dot-blot methodology (PCR dot-blot) and PCR agarose gel electrophoresis (PCR-AG) for the diagnosis of Pulmonary Tuberculosis (PTB) in HIV seropositive and HIV seronegative patients.

**Methods:**

A prospective study was conducted (from May 2003 to May 2004) in a TB/HIV reference hospital. Sputum specimens from 277 PTB suspects were tested by Acid Fast Bacilli (AFB) smear, Culture and *in house *PCR assays (PCR dot-blot and PCR-AG) and their performances evaluated. Positive cultures combined with the definition of clinical pulmonary TB were employed as the gold standard.

**Results:**

The overall prevalence of PTB was 46% (128/277); in HIV^+^, prevalence was 54.0% (40/74). The sensitivity and specificity of PCR dot-blot were 74% (CI 95%; 66.1%-81.2%) and 85% (CI 95%; 78.8%-90.3%); and of PCR-AG were 43% (CI 95%; 34.5%-51.6%) and 76% (CI 95%; 69.2%-82.8%), respectively. For HIV seropositive and HIV seronegative samples, sensitivities of PCR dot-blot (72% vs 75%; p = 0.46) and PCR-AG (42% vs 43%; p = 0.54) were similar. Among HIV seronegative patients and PTB suspects, ROC analysis presented the following values for the AFB smear (0.837), Culture (0.926), PCR dot-blot (0.801) and PCR-AG (0.599). In HIV seropositive patients, these area values were (0.713), (0.900), (0.789) and (0.595), respectively.

**Conclusion:**

Results of this study demonstrate that the *in house *PCR dot blot may be an improvement for ruling out PTB diagnosis in PTB suspects assisted at hospitals with a high prevalence of TB/HIV.

## Background

Tuberculosis (TB) is a persistent health problem, being responsible for 9.2 million cases per year. When associated with human immunodeficiency virus (HIV), TB is one of the leading infectious agents of death [1,2]. Frequently, the diagnosis of TB is based on the positive Acid Fast Bacilli (AFB) smear for Ziehl-Neelsen (ZN) staining, and this method detects around 70% of cases [2]. In clinical practice, the proportion of positive AFB smears is around 40-60% [3]. Usually, HIV seropositive patients demonstrate AFB smear negative staining for Ziehl-Neelsen (ZN) and present lower yields in this test for TB diagnosis. Moreover, these patients often present more atypical radiological findings and a higher mortality rate. The usual laboratory procedure for clinical specimens involves microscopic examination for the presence of AFB and isolation and identification of the organism by culture. In paucibacillary infections, the current detection method is culture, which can take up to six weeks until conclusion, due to the slow growth rate of mycobacteria. Timely identification of mycobacterial infection in HIV seropositive patients is critical to initiate early specific treatment, to improve prognosis and to reduce the risk of dissemination and spread to other hospitalized patients[4]. Therefore, a global strategy for the development and strengthening of laboratory diagnosis is urgently needed to improve the case detection rate, especially in regions with high prevalence of TB and HIV.

In recent years, rapid diagnostic tests based on nucleic acid amplification (NAA) tests have been developed [5,6]. In industrialized nations, automated NAA commercial tests are currently being used for the detection of *M. tuberculosis *complex organisms in respiratory specimens from adult patients, HIV seronegative and non-previously treated for TB[7].

Potential NAA techniques have been evaluated in developing countries, as these methods are more affordable; these *in house *methods frequently use the IS*6110 *element[7-13]. Accordingly, we evaluated the performance of two *in house *PCR methods: PCR dot-blot (colorimetric) and PCR-AG (non-colorimetric), using the IS*6110 *element as a target, for the diagnosis of Pulmonary Tuberculosis (PTB). We compared the status of HIV and the history of anti-TB treatment, in a setting of high prevalence of TB and HIV. This study was conducted according to routine procedures at the Reference Hospital of TB/HIV of a Southern Brazilian city, Porto Alegre.

## Methods

### Study location and population

Porto Alegre, a southern Brazilian city, had a population of 1,404,670, when the study was developed in 2004. Its public health system includes eight community health centers (CHC), 30 general hospitals, 10 specialized hospitals for pulmonary disease diagnosis and treatment and 3 hospitals based on correctional facilities. The Parthenon Reference Hospital (PRH) is the largest TB/HIV Reference Hospital and cares for both inpatients and outpatients. In 2004, in Porto Alegre City, 1432 cases of TB were reported. Among them, 201 (20%) were TB/HIV cases. These patients were assisted at CHCs and 213 (51%) at public hospitals[14].

### Design

A prospective study was conducted to evaluate the performance of two molecular tests for PTB diagnosis.

### Eligible and Ineligible Patients

PTB suspect patients, older than 18 years, assisted at PRH from May 2003 to May 2004 were eligible. Eligible patients were those: (1) who reported more than 3 weeks of cough. Patients ineligible were those receiving anti-TB treatment when they were asked to participate in the study. Patients with a history of previous TB were not excluded. Patients were excluded from the study if any of the following conditions were met: (1) culture was contaminated; (2) when expectorated sputum was not obtained (3) laboratory or clinical data did not fulfill the PTB definition; (4) written informed consent was not obtained from the study participant. All clinical samples were sent to the Laboratory of the State of RS, State Foundation for Research in Health, Porto Alegre/RS/Brazil, (FEEPS/Lacen/RS) for laboratory analysis. This study was approved by the Institutional Review Boards of FEPPS/RS (n. 01/2002).

### Logistics

PTB was diagnosed using a sputum specimen and was collected according to WHO recommendations[2]. The selection of the TB suspects entering the diagnostic process followed strictly routine diagnostic procedures of the Hospital. The local site coordinator was responsible for collecting all epidemiological data (patient interview was conducted with a validated questionnaire) and all specimens were sent to the Public State Laboratory, for laboratory analysis. Pneumologists were blinded to PCR results for the assessment of PTB cases, and laboratory technicians were also blinded to the clinical TB status of the clinical samples.

### Clinical Methods

Clinical PTB was defined by pneumologists using the clinical follow-up (symptoms, risk factors and chest X-Ray). Assessment of PTB suspect was undertaken during return visits by patients to the hospital and by the review of medical records respectively 6 and 12 months post diagnosis. Chest X-Ray was taken for those suspects whose symptoms were compatible with active TB and/or whose sputum smear AFB results were negative.

Identification of individuals who had had PTB in the past was defined as when the patient, during interview, related the previous use of anti-TB treatment for more than 30 days. Non-treated PTB was defined as those patients who were undergoing treatment for less than 14 days at the time of enrollment.

### Routine laboratory process and performance evaluation

All clinical samples were sent to the Laboratory of the State of RS, State Foundation for Research in Health, Porto Alegre/RS/Brazil, for laboratory analysis. AFB smear and culture assays were performed in the Culture Laboratory and PCR assays were performed in the Molecular Laboratory. All sputum samples were processed by the acetylcysteine method. AFB smear staining, according to the Ziehl Neelsen method, and culture were performed in Lowenstein Jensen method and identified according to Kubica's method[15].

### PCR methods

The presence of the amplified fragment of the IS*6110 *insertion sequence in positive PCRs was checked by electrophoresis with a 2% agarose gel, stained with ethidium bromide, and visualized under ultraviolet light [13]. The positive and negative controls were included in the electrophoresis analysis.

The PCR colorimetric dot-blot assay was performed, as previously published [13]. The DNA extraction from sputum was performed as previously published [12]. DNA was amplified by *in house *PCR using the IS*6110 *element as target, utilizing biotinylated primers to amplify a 132-bp DNA sequence specific to the *M. tuberculosis *complex SK1 (5'-AACGGCTGATGACCAAACTC-3') and SK2 (5'-GGTTAGGTGCTGGTGGTCC-3')[13]. The primers were synthesized by Invitrogen (Molecular Biology Incorporating Life Technologies™and ResGen™Brand). PCR products were purified in accordance with a description by Sperhacke et al 2004 and was analyzed in parallel using two procedures: (1) electrophoresis on 2% agarose gel, using TBE (1 ×) buffer, stained with ethidium bromide and visualized by ultraviolet transilluminator and (2) transfer to a nylon membrane and hybridization, according to Sperhacke (2004).

Briefly, aliquots of the amplified products were spotted. The amplified product was spotted on a nylon membrane (Biodyne B Gibco-BRL) in holes of an adapted support of propylene. A circle was drawn and the specimens were spotted inside of this circle for detection with a biotinylated DNA probe. The probe used in hybridization was obtained by amplification with the INS-1 primers (5'-*CGTGAGGGCATCGAGGTGGC *-3') and INS-2 (5'-*GCGTAGGCGTCGGTGACAAA *-3'). The detection of hybridization was performed using a conjugated streptavidin-alkaline phosphatase probe. The positive reaction was obtained by adding BCIP and NBT (; 5-bromo-4-chloro-3-indoyl phosphate and nitro blue tetrazolium; Sigma^®^). The positive and negative controls were included for each set of PCR A negative control (PCR mixture with water instead of template DNA), and positive control (*M. tuberculosis *Mt H37Rv, 100 ng) were included for each set of PCR. To detect specimen inhibitors, a duplicate tube of 50 μL PCR mix for each specimen was spiked with 2 μL of an aqueous solution containing 10 pg of purified DNA target [13]. All PCR tests with discrepancies in results were tested in duplicate. To avoid cross-contamination an extraction-negative control (a tube containing no organisms) and an extraction-positive control (a dilution of *M. tuberculosis *Mt H37Rv bacilli containing 50 colony forming units [CFU]) were included for each set of extractions.

### HIV

Blood samples were tested for HIV1 and HIV2 by serology (GenScreen HIV Plus^® ^BioRad), according to the manufacturer's instructions, and positive tests were confirmed by Western blotting (Genelabs^® ^Diagnostics).

### Ethics

This study was approved by the Institutional Review Boards of FEEPS (n. 01/2002).

### Gold Standard

Positive bacteriological result (at least one positive culture and biochemical identification) combined with diagnosis of clinical PTB.

### Independent Review

Two independent experts in TB diagnosis who did not participate in the study reviewed clinical PTB. In the absence of a consensus, a third TB expert was invited to consider whether the patients with discordant results would be considered to be free of TB or not.

### Analysis

Epidemiological and laboratory data were stored in a computer database and analyzed by appropriate statistical software (SPSS 10^®^). The accuracy, sensitivity and spectivitiy of both PCR methods was compared to the gold standard. The negative predictive value (NPV) was calculated using the following formula = SP _test _× (1-Prevalence)/(1-SE _test_) × Prevalence+ SP _test _× (1-Prevalence). We used the TB prevalence identified in the current study. The 95% confidences Intervals were calculate using appropriate statistical software (Epi info versão 6.04^®^). The area under the Receiver operating characteristic (ROC) curve, known as the AUC, was used to estimate the accuracy of diagnostic tests. Using a dichotomous predictor, AUC will measure the average of sensitivity and specificity.

## Results

### Study population

A total of 277 PTB suspect patients were enrolled. Prevalence of PTB was 46.2% (128/277); no history of prior TB treatment was reported by 73.3% (203/277), and prevalence of HIV infection was 26.7% (74/277). The prevalence of PTB among HIV seropositive subjects was 54.0% (40/74). Some risk factors for PTB were significantly more frequent in HIV seropositive patients than HIV seronegative patients: alcohol addiction (44.0% vs 25.1%; p = 0.002); TB in the past (56.2% vs 23.1%, p = 0.0007), previous hospital admission (41.3% vs 25.6%, p = 0.01), and schooling of less than 8 years (72.0% vs 58.6%, p = 0.04) (Table 1). Weight loss was observed more frequently among HIV seropositive individuals, 75.7%. The most consistent predictor of PTB in all patients was suggestive chest radiography (R: 0.36; p < 0.05), but in HIV seropositive patients, this predictor was not significant (R: 0.85; p = 0.32).

**Table 1:** Patient symptoms and medical history, according to HIV status

	HIV seronegative group			HIV seropositive group		
**Symptoms and** **Medical History**	**Overall suspects** **N = 203** **(%)**	**Non previously treated** **TB suspects** **N = 156** **(%)**	**TB in the past** **N = 47** **(%)**	**Overall suspects** **N = 74** **(%)**	**Non previously treated** **TB suspects** **N = 33** **(%)**	**TB in the past** **N = 27** **(%)**

**Positive Culture**	75 (36.9)	67 (42.9)	8 (17.0)	32 (43.2)	27 (81.8)	5 (18.5)

**Weight loss^a^**	104 (51.2)	79 (50.6)	25 (53.2)	56 (75.7)	36 (75.0)	20 (74.1)

**Cough**	190 (93.5)	148 (94.9)	25 (53.2)	67 (90.9)	43 (89.6)	24 (88.9)

**Chest pain**	121 (59.6)	96 (61.5)	31 (66.0)	42 (56.7)	22 (66.7)	14 (51.9)

**Dyspnea**	123 (60.1)	92 (58.9)	50 (67.6)	50 (67.6)	33 (100.0)	17 (63.0)

Comparison between HIV seropositive vs HIVseronegative ^a^p = 0.004

Chest X-Ray suggestive of classical tuberculosis (upper-lobe fibrocavitary) was observed more frequently in HIV seronegative (67.3%) than in HIV seropositive individuals (32.2%) (data not shown).

### Comparative performances of AFB smear, culture and two *in house *PCR methods in patients with or without a prior history of TB treatment, evaluated for PTB diagnosis

Overall, AFB smear sensitivity was 60% (CI 95%; 51,5%- 68.4%). PCR dot-blot sensitivity was [74% (CI 95%; 66.1%-81.2%)], which was significantly higher than that of PCR-AG sensitivity [43% (CI 95%; 34.6%-51.7%)]. The negative predictive value (NPV) of PCR dot-blot [81% (CI 95%; 72.6% - 85.1%)] was similar to that of the NPV of culture [88% (CI 95%; 82.0% - 91.9%)]; p = 0.067 (Table 2).

**Table 2:** Comparative performance of AFB Smear, Culture and two *in house *PCR dot-blot methods in PTB suspects

Laboratory Results and Performance of methods		All Groups^a^ N = 277				TB non-treated Group^b^ N = 203				TB in the past Group^c^ N = 74			
		
		TB N = 128		Non-TB N = 149		TB N = 109		Non-TB N = 94		TB N = 19		Non-TB N = 55	
Performance of AFB smear	Positive	77		1		68		0		9		1	
	
	Negative	51		148		41		94		10		54	

		SE (%)	SP (%)	PPV (%)	NPV (%)	SE (%)	SP (%)	PPV (%)	NPV (%)	SE (%)	SP (%)	PPV (%)	NPV (%)
		
		60	99	99	74	62	100	100	70	47	98	90	84

CI 95(%)		51.5-68,4	96,7-99,9	93.8-99.9	67,9-80,0	53,0-71,1	96,8-100	95,7-100	61,5-76,9	26,1-69,4	91,3-99,9	59,6-99,5	73,9-91,8

Performance of Culture	Positive	107		0		94		0		13		0	
	
	Negative	21		149		15		94		6		55	

		SE (%)	SP (%)	PPV (%)	NPV (%)	SE (%)	SP (%)	PPV (%)	NPV (%)	SE (%)	SP (%)	PPV (%)	NPV (%)
		
		84	100	100	88	86	100	100	90	68	100	100	90

CI 95(%)		76,4-89,3	98,0-100	97,2-100	82,0-91,9	78,8-91,8	96,8-100	96,8-100	78,8-91,8	45,5-86,1	94,7-100	79,4-100	80,7-95,9

Performance of PCR dot-blot	Positive	95		22		83		13		12		9	
	
	Negative	33		127		26		81		7		46	

		SE (%)	SP (%)	PPV (%)	NPV (%)	SE (%)	SP (%)	PPV (%)	NPV (%)	SE (%)	SP (%)	PPV (%)	NPV (%)
		
		74	85	81	81	76	87	86	76	63	84	57	86

CI 95(%)		66,1-81,2	78,8-90,3	73,3-87,5	72,6-85,1	67,5-83,4	78,0-92,1	78,5-92,2	66,9-83,1	40,3-82,2	72,1-91,7	35,7-76,7	75,6-94,0

Performance of PCR-AG	Positive	55		35		46		27		9		8	
	
	Negative	73		114		63		67		10		47	

		SE (%)	SP (%)	PPV (%)	NPV (%)	SE (%)	SP (%)	PPV (%)	NPV (%)	SE (%)	SP (%)	PPV (%)	NPV (%)
		
		43	76	61	61	42	71	63	51	47	85	53	82

CI 95(%)		34,6-51,7	69,2-82,8	50,8-70,5	53,8-67,8	33,2-51,4	61,5-79,7	51,5-73,5	42,9-60,0	26,1-69,3	74,2-93	29,7-75,2	70,9-90,7

SE: Sensitivity, SP: Specificity, PPV: Positive Predictive Value, NPV: Negative Predictive Value

**^a^**All group

**^b^**TB non-treated Group

**^c^**TB in the past Group

AFB smear and culture sensitivities were slightly higher among those not previously treated by TB than those observed among patients treated for TB in the past (62% vs 47%; p = 0.16), (86% vs 68%; p = 0.06), respectively. PCR dot blot specificity among those not previously treated was similar to that observed in patients treated for TB in the past (87% vs 84%; p = 0.42) and was slightly higher than PCR-AG specificity for not previously treated TB (87% vs 71%; p = 0.36), respectively (Table 2).

Among PTB suspects, AFB smear negative results were found in 71.8% (199/277). Of these individuals, in non-previously treated patients, PCR dot-blot had a sensitivity of 68% (CI 95%: 52.9%-81.0%) (data not shown).

### Comparative performances of AFB smear, culture and two in house PCR methods in patients evaluated for PTB diagnosis, according to HIV status

The AFB smear sensitivity was significantly lower in the HIV Seropositive group than in HIV seronegative individuals (43% for HIV seropositive and 68% for HIV seronegative; p < 0.05). In the HIV seronegative group, the AFB smear sensitivity was higher among non-previously treated patients than in those treated in the past, respectively (70% and 54%; p < 0.05); in the HIV seropositive group, there was no statistical difference among these groups (Table 3).

**Table 3:** Comparative performance of AFB Smear, Culture and two *in house *PCR dot-blot methods stratifying by HIV status

Laboratory Results and Performance of methods		HIV seronegative Group^a^ N = 203				HIV seropositive Group^b^ N = 74			
		
		TB N = 88		Non-TB N = 115		TB N = 40		Non-TB N = 34	
Performance of AFB smear	Positive	60		1		17		0	
	
	Negative	28		114		23		34	

		SE (%)	SP (%)	PPV (%)	NPV (%)	SE (%)	SP (%)	PPV (%)	NPV (%)
		
		68	99	98	81	43	100	100	60

CI 95(%)		57,9-77,2	95,8-99,9	92,2-99,9	73,2-86,2	28,0-58,0	91,6-100	83,8-100	46,6-71,7

Performance of Culture	Positive	75		0		32		0	
	
	Negative	13		115		8		34	

		SE (%)	SP (%)	PPV (%)	NPV (%)	SE (%)	SP (%)	PPV (%)	NPV (%)
		
		85	100	100	90	80	100	100	81

CI 95(%)		76,3-91,5	97,4-100	96,1-100	83,7-94,2	65,5-90,3	91,6-100	91,0-100	67,0-90,7

Performance of PCR dot-blot	Positive	66		17		29		5	
	
	Negative	22		98		11		29	

		SE (%)	SP (%)	PPV (%)	NPV (%)	SE (%)	SP (%)	PPV (%)	NPV (%)
		
		75	85	79	82	72	85	85	72

CI 95(%)		65,2-83,2	77,1-90,9	69,8-87,2	74,0-87,8	57,2-84,6	70,4-94,4	70,4-94,4	57,2-84,6

Performance of PCR-AG	Positive	38		27		17		8	
	
	Negative	50		88		23		26	

		SE (%)	SP (%)	PPV (%)	NPV (%)	SE (%)	SP (%)	PPV (%)	NPV (%)
		
		43	76	58	64	42	76	68	53

CI 95(%)		33,1-53,7	68,1-83,6	46,2-69,9	55,5-71,5	28,0-58,1	60,2-88,4	48,1-83,8	39,1-66,6

SE: Sensitivity, SP:Specificity, PPV: Positive Predictive Value, NPV: Negative Predictive Value

**^a^**HIV seronegative Group

**^b^**HIV seropositive Group

As shown in Table 3, culture sensitivity and NPV results remained similar (80% and 81% in HIV seropositive and, 85% and 90% in HIV seronegative groups, p = 0.31), in the two groups; PCR dot-blot sensitivity was higher than PCR-AG for both HIV seropositive, (72% and 42%, p < 0.05); and HIV seronegative (75% and 43%, p < 0.05) groups. NPV of PCR dot-blot was slightly lower for HIV seropositive individuals (72%), in comparison to HIV seronegative individuals (82%). Additionally, NPV of the PCR dot-blot (72%) was similar to that observed with culture (81%) in the HIV seropositive group (p = 0.54).

In HIV seronegative patients, not previously treated for TB, PCR dot-blot sensitivity was higher than that observed for those treated in the past (74% vs 40%, p > 0.05); but was not observed in HIV Seropositive individuals (Data not shown).

In smear negative PTB suspects, according to HIV status; PCR dot-blot had similar sensitivities (61% for HIV Seropositive and 64% for HIV Seronegative, p = 0.12) and specificities (85% HIV Seropositive and 85% HIV Seronegative, p = 0.10), respectively (Data not shown).

### Comparative estimate risk of correct diagnostic (*odds ratio-OR*) using of AFB smear, culture and two in house PCR methods

The risk of correct diagnostic (*odds ratio-OR*) was estimated, in overall patients the *OR *were 3.8 (CI 95%; 3.0 - 4.9) to AFB smear, 8.1 to Culture (CI 95%; 5.4-12.0), 1.6 to PCR-AG (CI 95%; 1.2 - 1.9)] and 3.9 to PCR dot-blot (CI 95%; 2.9 - 5.4)].

Among those not previously treated by TB the *OR *were to 3.3 (CI 95%; 2.5 - 4.2) to AFB smear, 7.3 to Culture (CI 95%; 4.5-11.6), 1.3 to PCR-AG (CI 95%; 1.0 - 1.7)] and 3.6 to PCR dot-blot (CI 95%; 2.5 - 5.0)].

However among HIV seropositive group the *OR *were to 2.5 (CI 95%; 1.8 - 3.4) to AFB smear, 5.2 to Culture (CI 95%; 2.8-9.8), 1.4 to PCR-AG (CI 95%; 0.97 - 2.2)] and 3.1 to PCR dot-blot (CI 95%; 1.8 - 5.2)].

### Inhibition and detection limit of two *in house *PCR

The inhibition of two *in house *PCR was 1.9%. Twenty-three specimens presented less than 50 CFU in culture (detection limit of *in house *PCR). These specimens were included in the analysis. Among these cases: 7 (30%) showed chest X-Rays suggestive of classical Tuberculosis, 14 (61%) presented weight loss, 3 (13%) hepatitis, 23 (100%) cough, 14 (61%) chest pain and 15 (65%) dyspnea.

### Comparison of accuracy of AFB smear, Culture, PCR dot-blot and PCR-AG tests using the area of ROC curve

**Figure 1:**
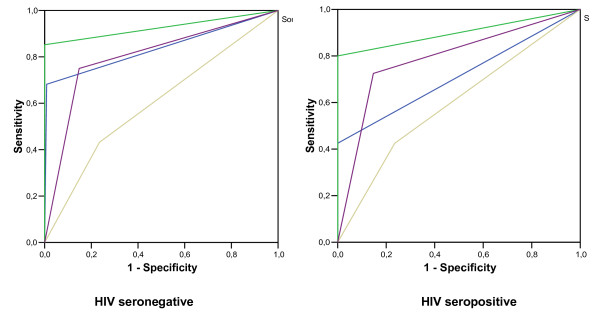
**ROC space Plot accuracy estimates for each method and areas corresponding to 203 HIV seronegative and to 74 HIV seropositive individuals**. (Blue: AFB Smear; Green: Culture; Yellow: PCR-AG and Purple: PCR dot-blot).

Among the 203 HIV seronegative patients and PTB suspects, ROC analysis showed the areas of AFB smear (0.837), culture (0.926), PCR dot-blot (0.801) and PCR-AG (0.599). Among the 74 HIV seropositive PTB suspects, the ROC areas were (0.713), (0.900), (0.789) and (0.595), respectively (Figure 1).

## Discussion

This study compared the performance of bacteriological and two *in house *PCR techniques for TB diagnosis in PTB suspects that were assisted at a TB/HIV Reference Hospital, using the first sample of expectorated sputum. The aim of this study was to employ techniques in a developing country with a large number of PTB suspects, evaluated for HIV status and previous anti-TB treatment. Patients were carefully characterized, with independent reviews to determine the final PTB cases.

We observed a high prevalence of active PTB (46.2%), and a high rate of HIV infection (27%) among PTB suspects, confirming the epidemiological data described by the Control Program of TB from Porto Alegre [14]. The most consistent predictor of PTB in all patients was suggestive of the chest X-Ray, but in HIV Seropositive patients this was not significant, and these patients frequently present more atypical radiological results[4]. Moreover, we observed a lower yield in the direct microscopy examination of expectorated sputum, as described previously[16,17]. These facts confirm that, in developing countries with a high prevalence of TB and HIV, better tests and more-efficient diagnostic processes are urgently needed[16].

Sensitivities of the PCR dot-blot, shown in Table 2, ranged from 63% to 76% and presented a trend towards higher sensitivity than that obtained with PCR-AG (42% compared to 47%). The PCR dot-blot sensitivities were statistically higher among non-previously treated patients, in comparison with those treated for TB in the past, despite the HIV status. Nevertheless, similar results were obtained with the AFB smear and culture, suggesting that in the non-previously treated group there was a higher bacterial load in the clinical specimens than in the group of patients treated for TB in the past. Among smear negative PTB suspects with or without HIV, the sensitivity of *in house *PCR (PCR dot-blot) ranged from 61% to 68%, similar to that reported in the meta-analysis of Sarmiento (32% to 92%), and also from studies carried out in developing nations using *in house *PCR techniques (40% to 64%), or using automated NAA tests (52% to 76%) [3,16,18-23]. Specificities of *in house *PCR ranging from 76% for PCR-AG to 87% for PCR dot-blot were similar to values described previously (77% to 92%) in developing countries, using automated NAA tests, and lower (>95%) than those described in industrialized countries[8,24-27].

Lower PCR-AG specificity (71%) among those patients that had not previously been treated could be due to contact with respiratory symptomatic patients; in fact among these patients with false positive results, 18 (67%) reported previous tuberculosis contact. Lower specificity of PCR dot-blot (84%) among those patients with anti-TB treatment in the past was found to occur in those patients with previous infection, thus it is not surprising that DNA could be detected from their respiratory specimens. Decreased specificity for PCR has also been reported in other studies using *in house *PCR tests [28,29]

The lower sensitivity of both *in house *PCR and PCR-AG (42%) among not previously treated patients and the lower sensitivity of PCR dot-blot (63%) among patients with previous anti-TB treatment may be due, in part, to the presence of inhibitors that remain in the specimen following the current extraction procedure and/or a small number of mycobacteria that were unequally distributed in test suspension or below the detection limit of the amplification of this test (50 CFU)[13]. In fact, in our study, among false negative results, 20 (32%) in PCR-AG and 3 (43%) patients in PCR dot-blot, were below the detection limit of the amplification test. The proportion of inhibitors was (1.9%) for *in house *PCR, similar to the studies using automated NAA (0.85% to 5%) and lower than those of other reports that used *in house *PCR (3.7% to 22.7%)[8,27,30,31]. The use of the IS*6110 *insertion element as the PCR target could be a potential source of decreased sensitivity, since MTB lacks this element, as previously reported. However, DNA fingerprinting studies performed in Brazil and especially in our state (RS), did not detect the presence of these strains. On the contrary, the great majority of strains presented high copy numbers of IS*6110 *[32,33]

## Conclusions

In this report, the sensitivity of the AFB smear was significantly lower in HIV seropositive/TB patients, and the sensitivity of both *in house *PCRs was not influenced by the HIV status, similar to data reported by others [16,17].

In the present study, the analysis of the plot in the ROC space of accuracy in all patients shows a similar performance for culture and PCR dot-blot in HIV Seropositive and HIV-PTB suspects. The culture method showed the best performance for PTB diagnosis; however, more than 6 weeks are necessary to obtain the final result. Fast identification of mycobacterial infections is necessary, especially in HIV/TB patients, who need an early appropriate and specific treatment to improve prognosis.

Possible study limitations of the study were the use of only one respiratory specimen (which can lead to a lower sensitivity) instead of two or three specimens for outpatients, as proposed by WHO. However, we analyzed outpatients and inpatients; rapid diagnosis of PTB is important for these patients and it is sometimes difficult to obtain three specimens, particularly in TB/HIV patients. Other limitations were the presence of inhibitions of *in house *PCR and the low limit of detection of 50 CFU. These findings may influence the performance of PCR tests[3,7]. In fact, laboratory studies have suggested low sensitivities of PCR for the diagnosis of PTB and the significant variability in sensitivities and specificities in different studies, mainly due to the decontamination procedures, cross contaminations, sampling error inhibitions, detection limit of tests and quality of the reference standard [3,30,34].

Although the information in a diagnostic test can be summarized using sensitivity and specificity, other parameters may be clinically important for the definition of the accuracy of a laboratory test. The positive predictive value (PPV) is the proportion of true positives in all positive results and shows the probability that one patient with a positive test has the disease. The negative predictive value (NPV) is the proportion of true negatives in all negative results and shows the probability that one patient with a negative test does not have the disease. However, these parameters are dependent of prevalence rate. So for different prevalence rates can be found different predictive values (NPV and PPV). The predictive values showed in our setting with 46,2% of TB prevalence, should be interpreted with attention and prevalence of other settings should be considered.

The predictive values (PV) for different prevalence rates could be calculate using specific formulas: PPV = SE_test _× Prevalence/(SE _test _X Prevalence) + (1-SP _test_) × (1-Prevalence) and NPV = SP _test _× (1-Prevalence)/(1-SE _test_) × Prevalence+ SP _test _X (1-Prevalence)

Othe parameter utilized for the definition of teh accuracy of a laboratory tests is a ROC curve. ROC curve analysis is a technique for assesing diagnostic tests, based on the notions of specificity and sensivity, which can be used to evaluate the accuracy of tests and also to assess predictive models. We used this technique to evaluate the accuracy of tests through of AUC.

The risk of correct diagnostic (*OR*) was higher than Culture than others methods. However in patients HIV seropositives the PCR dot-blot was similar to Culture, confirming that this technique can be usefulness to correct diagnosis of PTB.

This study shows that *in house *PCR, using a colorimetric system of revelation, may offer an improvement for ruling out PTB diagnosis, for PTB suspects not treated previously, evaluated in hospitals, and in areas with high prevalence of TB and HIV. Of the *in house *PCR tests, PCR dot-blot seems to be more appropriate for routine use, since this method includes a hybridization step, which increases the sensitivity of detection. It also offers higher accuracy, rapidity, ease of use, greater safety, cost effectiveness and greater objectivity in the reading of results, as reported previously[35].

Additionally, *in house *PCR tests are usually less costly than automated NAA and might be introduced more widely after a proper evaluation in different settings of its clinical utility and cost-effectiveness.

## Competing interests

The authors declare that they have no competing interests.

## Authors' contributions

LCS carried out the study, participated in the laboratory tests, participated in data acquisition, performed the statistical analysis and drafted the manuscript; RDS carried out the laboratory tests, data analysis, participated in data acquisition and drafted the manuscript, RR participated in data acquisition and drafted the manuscript, CJ participated in data acquisition and drafted the manuscript, PIC carried out the laboratory tests, data analysis, participated in data acquisition and drafted the manuscript, CM carried out the laboratory tests, data analysis, participated in data acquisition and drafted the manuscript, MOR carried out the laboratory tests, data analysis, and drafted the manuscript, ARN performed the epidemiological analysis and drafted the paper, MLRR helped design the study, performed the statistical analysis and drafted the paper, CJ helped to draft the manuscript, ALK conceived the study, participated in its design, performed the data analysis, coordination and helped to draft the manuscript. All authors contributed to the interpretation of results and have read and approved the final manuscript.

## Pre-publication history

The pre-publication history for this paper can be accessed here:

http://www.biomedcentral.com/1471-2466/11/15/prepub
